# Social problems in oncology

**DOI:** 10.1038/sj.bjc.6600642

**Published:** 2002-11-04

**Authors:** E P Wright, M A Kiely, P Lynch, A Cull, P J Selby

**Affiliations:** Cancer Research UK Clinical Centre, St James's University Hospital, Leeds, UK; Cancer Research UK, Medical Oncology Unit, Western General Hospital, Edinburgh, UK

**Keywords:** social problems, cancer, focus groups

## Abstract

A study was undertaken to describe, evaluate and categorise the social problems experienced by cancer patients. Ninety-six adult cancer patients at all stages of disease participated in either a telephone focus group discussion, a face to face focus group or an individual interview which were tape recorded and transcribed. Six experts analysed the transcripts. A total of 32 social problems were identified categorized under eight headings plus four single items. The categories were: problems with (1) managing in the home, (2) health and welfare services, (3) finances, (4) employment, (5) legal matters, (6) relationships, (7) sexuality and body image and (8) recreation. Problems with relationships and communication were the most frequently reported with financial, employment, body image and domestic problems also being widely endorsed. Female groups, younger patient groups and groups where the aim of treatment was palliative reported more social problems than other groups. Social problems are common and important to cancer patients. The social problems identified in this study will contribute to an item pool generated for developing a Social Problems Inventory that may be included in patient centred assessment as part of routine oncology practice.

*British Journal of Cancer* (2002) **87**, 1099–1104. doi:10.1038/sj.bjc.6600642 www.bjcancer.com

© 2002 Cancer Research UK

## 

Science has dominated medicine for the last century and this has served oncology patients well. Over the last three decades, following advances in cancer treatments and the introduction of screening for early detection of breast and cervical cancer, survival rates have improved markedly (Coleman *et al*, 1999). Therefore, for an increasing proportion of the cancer patient population survival becomes a way of life. This good news may be tainted with the burdens of toxicity, uncertainty, chronic disability and discrimination. Patients attend oncology clinics from a diversity of backgrounds and differing social histories. They belong to families, local communities and the wider world. Patients have responsibilities in the home, the workplace and recreationally. A cancer diagnosis may threaten any one of these arenas resulting in a range of potential social problems. There may be complex interactions between social problems which are a direct result of the cancer and its treatment and those social problems which are an underlying reflection of the life, social status or economic status of the individual patients.

Routine assessment of social problems is not part of standard oncology practice. The primary preoccupation of the oncology team will usually lay an emphasis on maintaining survival. This preoccupation is also of paramount importance to patients and patients' families. There is evidence, however, to suggest that, although many patients make a good adjustment to having had a cancer diagnosis and treatment (Zebrack, 2000), there are others who may struggle and who would benefit from psychosocial support (Cull *et al*, 1995). As services stand at present this aspect of care is often neglected as health care professionals have other priorities and patients may feel ungrateful if they make demands on a service that is focussing on maintaining survival.

Advances in biological science have not prevented psychosocial dimensions of cancer care from developing but they have relatively low priority in oncology and this may be particularly true of social problems. Lack of clear definitions and methods of measurement may account for this in part. Defining social problems is difficult and the range of potential problems enormous. However, the detection and characterisation of social problems may lead to an improvement in the care of cancer patients and result in enhanced patient well-being. Integrating assessment of social problems into routine oncology practice is a challenge.

The aim of this study was to discover the character, breadth and prevalence of social problems experienced by cancer patients by using patient focus groups and interviews.

## MATERIALS AND METHODS

### Sample

Following approval from the local ethics committees patients were recruited from the outpatients lists of two surgeons, eight clinical oncologists, four medical oncologists and two consultants in palliative medicine working over three hospitals and one hospice in Leeds. Patients who were deaf, had speech difficulties or were unable to speak English were excluded from the study.

At this time uncertainty over the interpretation of the recently implemented Data Protection Act 1998 lead to the sample being recruited by medical staff only and information not being collected on the group of patients who refused to take part in the study.

Participants were purposely selected for one of 18 telephone focus groups: by age (under 40 years, 40–60 years and over 60 years), by gender and by treatment stage (patients undergoing or recently completed treatment with the aim to cure, those being monitored or having maintenance treatment for stable disease/remission, and patients receiving palliative care/treatment), shown in Table 2

**Table 2:**
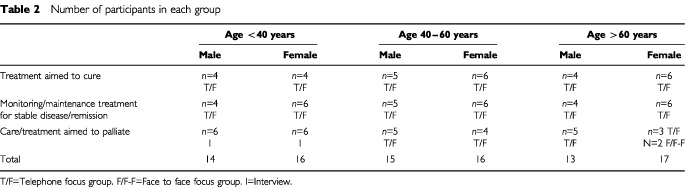
Number of participants in each group

. A maximum of six patients was recruited for each group. A face to face focus group was arranged for patients with head and neck cancer. Sixteen of the 18 telephone focus groups were run as planned. Two groups could not be scheduled. These were for the men and women under the age of 40 receiving palliative care. This was due to the participants' deteriorating health or their admission to hospital. As a result of postponement and continuing crises 12 patient face-to-face interviews were undertaken. A second focus group for women over 60 receiving palliative care was run at the local day hospice. This was due to the low participation rate in the telephone group following deterioration in health of women who had consented. The results of these two groups were pooled.

St James's University Hospital, Cookridge Hospital and Leeds General Infirmary are part of the Leeds Cancer Centre and are main teaching hospitals with a large cancer care catchment area. St Gemma's Hospice is one of two hospices in Leeds with 45 in-patient beds, an active day hospice and established community service.

### Procedure

The design, procedures and analysis plan of the focus groups were based on the guidance of Krueger (1994).

Patients were invited to participate by the medical team in outpatient clinics or by letter from their consultant and consenting patients were allocated to groups according to age, sex and treatment aim. Clinical and sociodemographic information was collected from the medical notes and patients. Groups were moderated by two of the research team using the method recommended by Krueger (1998a) and adapted for use over the telephone. An interview guide was designed (Table 1

**Table 1:**
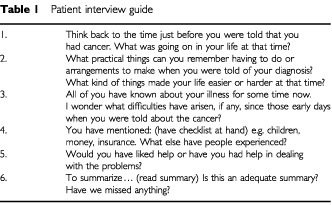
Patient interview guide

) incorporating general introductions followed by the question format advocated by Krueger (1998b).

### Telephone focus groups

The registered charity Community Network was used to facilitate the telephone conference call link up and tape recording of all the telephone focus groups. Costs were paid for directly by the research unit. Moderators were contacted first followed by the participants. All participants had an immediate follow up phone call for debriefing.

### Face to face focus groups

One group was run for head and neck cancer patients in a meeting room at a local research centre. The hospice day centre group met during their weekly attendance at the unit.

### Interviews

Eight interviews were carried out in the patients' homes, one at a place of work and three were undertaken in hospital.

All focus groups and interviews were audio taped and transcribed. Sessions lasted up to 1 h. Field notes of each session were made following the completion of each group. The same interview guide, other than an initial general introduction question, was used for the interviews and focus groups.

### Analysis

#### Coders

Six coders undertook the analysis of the transcripts. The coders were professionals from health and social care with expert knowledge of oncology. Krippendorf recommends that analysis of thematic units (of which this analysis is a simple adaptation) should be undertaken by people who have a depth of knowledge of the subject in order for the units to be identified reliably (Krippendorf, 1980; page 62).

### Framework for analysis

The experts met to agree a framework for analysis. Each transcript was coded by two experts independently of each other. Items were included in the analysis if they were defined as social problems (external difficulties). Psychological problems, disease symptoms and treatment side-effects were not included in the analysis unless they comprised aspects of social problems in addition to the other dimensions. Items were counted if reported as having been experienced by individual participants but not counted if non-specifically referred to in discussion. The analysis took the form of a simple number count (Krueger, 1998c). Item extensiveness (the number of groups relating the social problem) and item frequency (the number of times the social problem described was mentioned) were calculated (Krueger, 1998c). A mean frequency was calculated for each social problem within every transcript by summing the number of times that it was recorded by each coder and dividing the sum by two (mean frequency). From this simple count, items with similar characteristics were grouped under more general category headings.

### Stages of analysis

The analysis of the transcripts was undertaken in three stages. Stage one: PS and PW coded the first 10 focus group transcripts independently. Discovery was compared and a list of clearly defined social problem items generated. Stage two: the generated item list was discussed at a consensus meeting of the four other experts (MF, MK, SK and VW) and main researcher (PW). Definitions were clarified where uncertainty existed. The four experts were paired and each expert was given four of the next eight transcripts to rate. Additional previously unreported social problems were added if identified in the transcripts by any of the experts. Following the completion of the coding the four experts and PW held a consensus meeting and, where there was obvious disagreement over definition, further discussion followed until consensus was arrived at. An amended list of clearly defined social problems was generated. Stage three: the remaining 12 individual patient interview transcripts were coded by PW and MK. Coding was undertaken using the social problem item list derived from stage two. The number of new social problems coded was counted as the analysis progressed to ensure that ‘item redundancy’ (saturation) had been reached (Streiner and Norman, 1995, page 17).

### Reliability

Each coder recorded the presence or absence of each social problem in every transcript. Inter-rater reliability was assessed from stage two onwards, using clearly defined social problems agreed from the consensus meetings, by calculating percentage exact agreement between paired coders for each item over all transcripts. This was not calculated for the first 10 transcripts as both coders generated the list of items ‘cold’ and there was no consensus about definitions.

## RESULTS

### Participants

Two hundred and seventy-eight patients were invited to participate. Of these, 140 refused (50.4%) and 138 consented (49.6%). Ninety-six patients took part including five male head and neck cancer patients who took part in the face-to-face focus group (not shown in Table 2). The main reasons for non-participation of those who consented were inability to schedule their time to fit in with the rest of the proposed group, personal reasons or deteriorating health/admission to hospital. Breast, lung and testicular cancers were the most frequently occurring with 13 other sites of cancer represented including ovarian, pancreatic, head and neck, GI tract, brain, cervical and kidney cancers. Although up to six patients were recruited for each group the numbers who participated were less in some groups due to last minute alterations in plans including emergency admission to hospital (Table 2).

Forty-six men and 50 women participated, age range 21–88 years (mean 50.8, SD 16.41). Seventy of the participants were married or cohabiting. Twenty-five participants were caring for dependent children or adults. Thirty participants were retired, 41 were in full or part time employment of which 24.4% were not working and 17.1% were working fewer hours. Since the time of diagnosis the income of 37 participants had decreased.

### Social problems

The final social problem item list generated following all three stages of analysis comprised a total of 32 items (Table 3

**Table 3:**
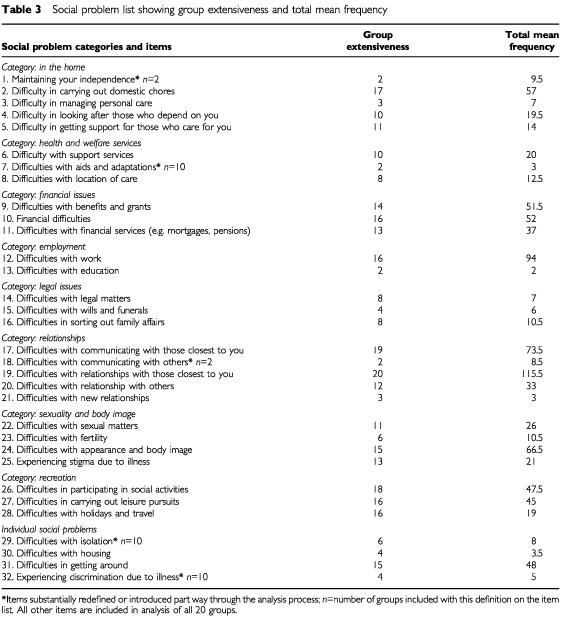
Social problem list showing group extensiveness and total mean frequency

). Items that gave rise to the most discussion concerning definition were those categorised under the general heading ‘relationships’ and the single item of ‘difficulties with getting around’.

The mean frequencies of recording each social problem were calculated within each transcript. These were summed resulting in a total mean frequency for each social problem. The number of groups recording the social problem was noted (group extensiveness) (Table 3).

**Figure 1:**
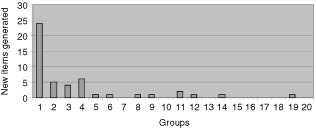
The number of new social problems generated by groups in order of analysis.

The most extensive and frequently coded social problems were those concerning relationships and communication with those close to the patient. Other issues of importance to the majority of patients groups were social activities and domestic chores. The number of new social problems generated from each group as the analysis progressed was counted (Figure 1

) in order to be confident that discovery of items was complete and that item saturation had been reached.

Inter-rater reliability was reasonable with 78% of the social problems identified by coders having percentage exact agreement scores of over 75%. Lower rates of inter-rater reliability were found in some transcripts for the following items: location of care, isolation, accessing support services, caring for dependents, legal issues and items under the relationship category.

### Categories of social problems

Group extensiveness scores were combined by age group, sex and stage of treatment to evaluate whether there were types of social problem common to specific groups. The head and neck patient group results were not incorporated into this analysis as the group comprised patients from different age groups and stages of treatment. ‘Maintaining your independence’, ‘aids and adaptations’, ‘isolation’ and ‘discrimination’, that is, items substantially redefined or introduced part way through the analysis process, were not included.

Eight key social problem categories were identified and two single social problems (Table 4

**Table 4:**
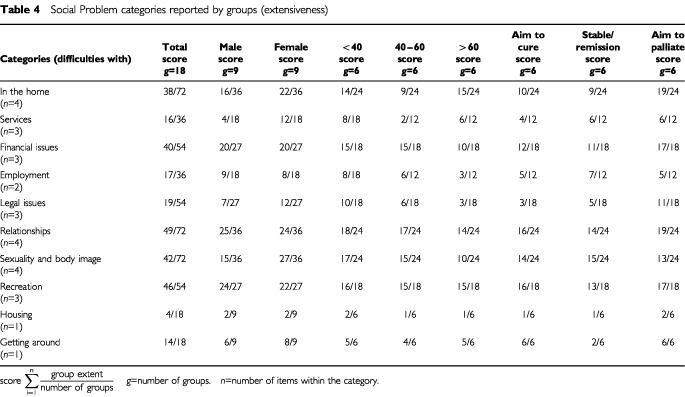
Social Problem categories reported by groups (extensiveness)

).

Female groups reported more items than male groups (55 *vs* 45%), the youngest age groups more than the older age groups (40 *vs* 31 and 29%) and those in the palliative care groups a greater number than those in the other treatment defined groups (40 *vs* 30 and 30%).

## DISCUSSION

This study describes the categories of social problems of our cancer patients and gives an indication of their prevalence. Social problems that stand out as being easily recognised are those where frequency and extensiveness values were high and inter-rater reliability was good. These social problems include (i) domestic chores, (ii) mobility, (iii) employment, (iv) benefits and finances, (v) mortgages, pensions and insurance (vi) social and leisure activities and (vii) body image. All of these, except body image, are easily defined clear concrete external difficulties. Body image is a little different in that it carries a greater measure of psychological content than the other items.

We have to be cautious in interpreting the prevalence data because only about half of patients consented. However the description of the categories and nature of the problems is robust in that we continued across a broad group of diagnoses and patient characteristics until no new items were identified (‘saturation’). The number of patients declining to take part in the patient study was high which was different to other studies within our psychosocial oncology research team where, on average, participation rates run at 70%. This study was started soon after the 1998 Data Protection Act was implemented. Uncertainty about how the Act would be interpreted led to caution in how patients were approached with the initial request not coming from the researchers but from their medical consultants, a practice not required in previous studies. Although the doctors who had responsibility of care for these patients were enthusiastic about the study, they may not have had the time to introduce the study to potential participants as carefully as could have been done by the researchers due to the multiple demands of their jobs. In addition to this restriction, apart from a simple number count, information could not be collected on the people who refused to participate. It is impossible to speculate whether the participators were in any way different from the patients who refused. The problem of accessing the full patient population has now been overcome at a local level and should not arise in future studies.

Thirty per cent of those who consented did not take part. This reflects the difficulties of attempting to fix times to run a group when many of the participants are liable to changes in medical plans, which are beyond their control. In a sense the difficulties experienced in setting up a focus group with cancer patients are similar to the difficulties experienced by cancer patients in forward planning social events. The groups that were hardest to organize were those with patients receiving palliative care. Many of these patients were very ill and had to withdraw before the group could be run. This was particularly noticeable with the younger age groups where fortunately advanced cancer is rare and therefore rate of recruitment slow due to lack of potential participants. However, when recruitment is very slow there is the danger of the patients recruited early on becoming too ill to participate by the time the numbers needed to run a group were consented. This was the case with this study and the 12 interviews resulted. However, over half of the social problems defined were reported by the majority of groups and the rate of item saturation suggests that it is unlikely that there were other important social problems that should have been elicited that were not realised, despite the fact that the participants had been drawn from a narrow base.

The telephone groups were simple to set up using Community Network and only on 2 out of 16 occasions was there a muddle with the order of link up. On both these occasions some of the participants were called prior to the moderator, causing some confusion and possibly influencing group dynamics. The feedback from the follow up debriefing calls was very enthusiastic. Initially some patients had expressed concern about the teleconferencing but were fully converted by the end of the session, wanting to take part again if the opportunity arose. This applied equally to male and female groups.

The range and depth of social problems reported by the patients clearly demonstrates that these issues are common and of importance.

Difficulties concerning relationships are high on the patient agenda, as was shown in the reported frequencies, but they are complex, vary enormously and cannot be easily categorised. There was much debate over the items concerning relationships in the patient focus group study and inter-rater reliability scores were only moderate. Attempts at reconciliation as the coding progressed were reflected in the final definition outcome of ‘relationships with those close to you’, ‘relationships with others’, ‘communication with those close to you’ and ‘communication with others’.

The experience of uncertainty over definition and difficulty with measurement of relationship problems was also described in the factor analysis of the Problem checklist where reliability was borderline for the factor labelled ‘relationships’ (Wright *et al*, 2001).

All categories of social problems were experienced by all groups although there were differences between the age groups, sexes and treatment stages experienced in the extensiveness and frequency of reported social problems. Women's groups disclosed more social problems than the men's groups. One explanation for this might concern changes in gender role. Women have moved into the traditionally male dominated arena of employment comprising, in this study, a similar proportion of full time workers as men. However, despite this shift, they do not appear to have given up their traditional role of primary homemaker and as a result may be burdened with a wider range of responsibilities than men. Similar findings have been reported in studies of unmet need in a general oncology population (Sanson-Fisher *et al*, 2000) and in investigation of gender differences in illness-related distress (Keller and Henrich, 1999). The study also found that men rely more on their wives for support whereas women turn to their family and friends rather than to their husbands.

The groups of under 40s reported the greatest number of social problems with the two older age groups endorsing a similar number to each other. This pattern has been observed in a study investigating psychosocial concerns (Harrison and Maguire, 1995) and one looking into unmet need (Mor *et al*, 1994). Younger people may have to cope with demands from a wide range of life domains including employment and child care which are less likely to be of significance to the older age groups. Older patients are more likely to suffer from co-morbid conditions (Coebergh *et al*, 1999) but despite this still report fewer problems. Elderly patients may make less of adverse circumstances and adapt better than younger people to restrictions induced by the cancer, accepting the change as par for the course.

Patients from the palliative care groups endorsed the greatest number of social problems, with groups from the aim to cure and stable disease/remission groups recording lower comparable figures. This corresponds quite closely to the prevalence of psychological distress (Zabora *et al*, 1997) and psychologic needs (Sanson-Fisher *et al*, 2000) found in similarly defined groups of patients. The high rates of endorsement from the palliative care groups may have been as a result of side-effects brought about by the burden of numerous treatments or from general debility induced by advanced disease.

Multiple social problems may make a contribution to increased levels of psychological distress. Alternatively, psychological distress may make social difficulties harder to cope with. Both views support the need for comprehensive assessment of social problems and psychological distress as part of patient centred care in all oncology practice. With advances in technology, notably the use of electronic methods of data collection, this type of patient centred assessment, including assessment of quality of life, psychological distress and social problems becomes possible (Newell *et al*, 1997; Velikova *et al*, 1999). We can now move towards developing a Social Problems Inventory (SPI), using the social problems elicited from this study along with items generated from health and social care staff and from the literature to form a basic item pool. Items concerning relationships, where reliability was only moderate and consensus difficult to achieve, must be subject to particular scrutiny during questionnaire construction and testing. Prior to any recommendations being made for including a Social Problems Inventory in a patient centred assessment reliability and validity of the instrument must be checked. This is being undertaken in a formal psychometric evaluation study.

Cancer patients are not unique. Social problems experienced by cancer patients may apply equally to other patient groups. The social and psychological aspects of patient care are at the heart of a patient centred health service. In this study the social problems of cancer patients have been highlighted. This endorses the need for assessment of social problems along with assessment of QL and psychological distress, in oncology clinics, which will enhance multidisciplinary care of cancer patients.
